# Effects of missing teeth and nasal septal deviation on maxillary sinus volume: a pilot study

**DOI:** 10.1186/s40729-022-00415-5

**Published:** 2022-04-15

**Authors:** Kikue Yamaguchi, Motohiro Munakata, Yu Kataoka, Takashi Uesugi, Yoshiaki Shimoo

**Affiliations:** 1 Department of Implantology Dentistry, Showa University School of Dentistry, 2-1-1, Kita-Senzoku, Ota-ku, Tokyo, 145-8515 Japan; 2 Division of Biomaterials and Engineering, Department of Conservative Dentistry, Showa University School of Dentistry, Tokyo, Japan

**Keywords:** Maxillary sinus augmentation, Maxillary sinus volume, Nasal septal deviation, Tooth loss

## Abstract

**Purpose:**

Tooth extraction and the projection of the tooth roots into the maxillary sinus are reported to greatly reduce the bone height from the alveolar ridge to the maxillary sinus floor, while missing teeth are reported to lead to the expansion of the maxillary sinus, all of which are important considerations during dental implant treatment for the maxillary molar region. Therefore, assessing the anatomical characteristics of the maxillary sinus acting as complicating factors is crucial before sinus augmentation. We conducted a three-dimensional examination of the effects of missing teeth and nasal septal deviation (NSD) on maxillary sinus volume (MSV).

**Methods:**

We selected participants with two or more missing teeth from patients who underwent maxillary sinus augmentation for a unilateral free-end saddle between April 2019 and December 2020. We calculated the MSV and NSD using cone-beam computed tomography (CBCT). We compared the relationships of the presence/absence of teeth and NSD with MSV bilaterally in each patient using the Wilcoxon *t*-test. *p*-values < 0.05 denoted statistical significance.

**Results:**

This study included 30 patients (30 sinuses; 12 men, 18 women). The average patient age was 58.2 ± 10.2 years (men, 60.4 ± 3.7 years; women, 59.2 ± 4.5 years; range, 40–77 years). The mean number of missing teeth was 2.98 ± 1.01: 13 patients had two missing teeth and 17 had three or more missing teeth. Nine patients (30%) had NSD. The mean MSV on the ipsilateral and contralateral sides of the NSD was 21.50 ± 3.84 cm^3^ and 22.10 ± 3.56 cm^3^, respectively; thus, NSD did not affect MSV (*p* = 0.150). The mean MSV on the edentulous and non-edentulous sides was 21.58 ± 3.89 cm^3^ and 21.77 ± 4.30 cm^3^, respectively; thus, the MSV was significantly smaller on the edentulous side (*p* = 0.00036).

**Conclusion:**

Although this study was a limited preoperative study, three-dimensional measurement of the maxillary sinus with CBCT in partially edentulous patients revealed that missing teeth lead to substantial reductions in MSV, while NSD was not associated with MSV.

## Background

The reason for tooth extraction and the projection of the tooth roots into the maxillary sinus is reported to greatly reduce the bone height from the alveolar ridge to the maxillary sinus floor, while missing teeth are reported to lead to the expansion of the maxillary sinus, both of which are important considerations during dental implant treatment for the maxillary molar region [1, 2]. Consequently, patients often require maxillary sinus augmentation, which was extremely successful in 94.9–100% of cases by a systematic review, irrespective of the implant material used [3]. Thus, maxillary sinus augmentation has become a widespread treatment option with long-term stability. However, several studies have reported intraoperative and postoperative complications, such as arterial injury, sinus membrane perforation, and sinusitis [3–5].

As anatomical risk factors for intraoperative complication, such as maxillary sinus membrane perforation, Testori et al. reported sinus membrane thickness, the presence of sinus septa, palatonasal recess, and sinus contour [6], while Akay et al. [7] and Lee et al. [8] reported on the relationship between sinus volume and the presence of septum and sinus membrane thickness. In another study, Testori et al. reported that sinus membrane thickness, nasal septal deviation (NSD), previous history of sinusitis, and natural ostium obstruction were anatomical risk factors for postoperative maxillary sinusitis, while the anatomical structure of the maxillary sinus is known to affect intraoperative and postoperative complications [9]. Moreover, the osteomeatal complex (OMC), composed of structures, such as the nasal septum, concha bullosa, middle nasal concha, uncinate process, and Haller cells, reportedly affects sinus membrane thickness which is a risk factor for maxillary sinus membrane perforation and postoperative maxillary sinusitis [9]. Another study reported that the maxillary sinus volume (MSV) differs between individuals with and without maxillary sinusitis [10]. Furthermore, the relationship between sinus volume and polyp formation and chronic rhinosinusitis has also been reported, suggesting a strong association between sinus volume and maxillary sinus lesions and OMC [11]. Thus, assessing the anatomical characteristics of the maxillary sinus that function as complicating factors in maxillary sinus augmentation is considered absolutely crucial.

However, most studies on the anatomical characteristics of the maxillary sinus have conducted two-dimensional assessments based on the sagittal or coronal plane of computed tomography (CT). Few studies have conducted three-dimensional examinations of missing teeth and the OMC, and none have performed comparisons within individual patients.

Therefore, we conducted a three-dimensional examination of the effects of missing teeth and NSD on MSV.

## Methods

### Study design and participants in this prospective study

We selected patients with two or more missing teeth from among those who underwent maxillary sinus augmentation for a unilateral free-end saddle at Showa University Dental Hospital Department of Implant Dentistry between April 2019 and December 2020.

### Eligibility criteria

The inclusion criteria were as follows: (1) cone-beam CT (CBCT) performed ≥ 3 months after tooth extraction; (2) patients aged > 40 years; (3) lateral missing molars, (4) residual bone height in the loss area < 4 mm; and (5) presence of partial edentulism in the posterior region of the maxilla.

The exclusion criteria were as follows: (1) maxillary sinus floor mucosal thickness ≥ 5 mm on CBCT; (2) smoking; (3)with a septum; (4) presence of non-transparent objects in the maxillary sinus, such as mucinous cysts or polyps; (5) history of paranasal sinus disease; (6) bronchitis, bronchial asthma, or other respiratory illnesses; (7) anticoagulant therapy for conditions, such as ischemic heart disease; (8) uncontrolled diabetes; and (9) periapical inflammatory disease in the maxillary posterior teeth.

Due to the lack of similar research and preliminary outcome studies, we chose a sample size of 30 as adequate. Power study indicated that a sample size of 25 would result in an 80% chance of detecting a meaningful effect (significance *p* < 0.05 [two-sided test]; 0.6 effect size).

The study protocol conformed to the ethical principles established during the World Medical Association Declaration of Helsinki of 1975 as revised in 2000. This study was approved by the Ethical Committee of Showa University Dental Hospital (Approval Number: DH2020-020).

### Measurements

#### MSV based on CBCT data measurements

**Fig. 1:**
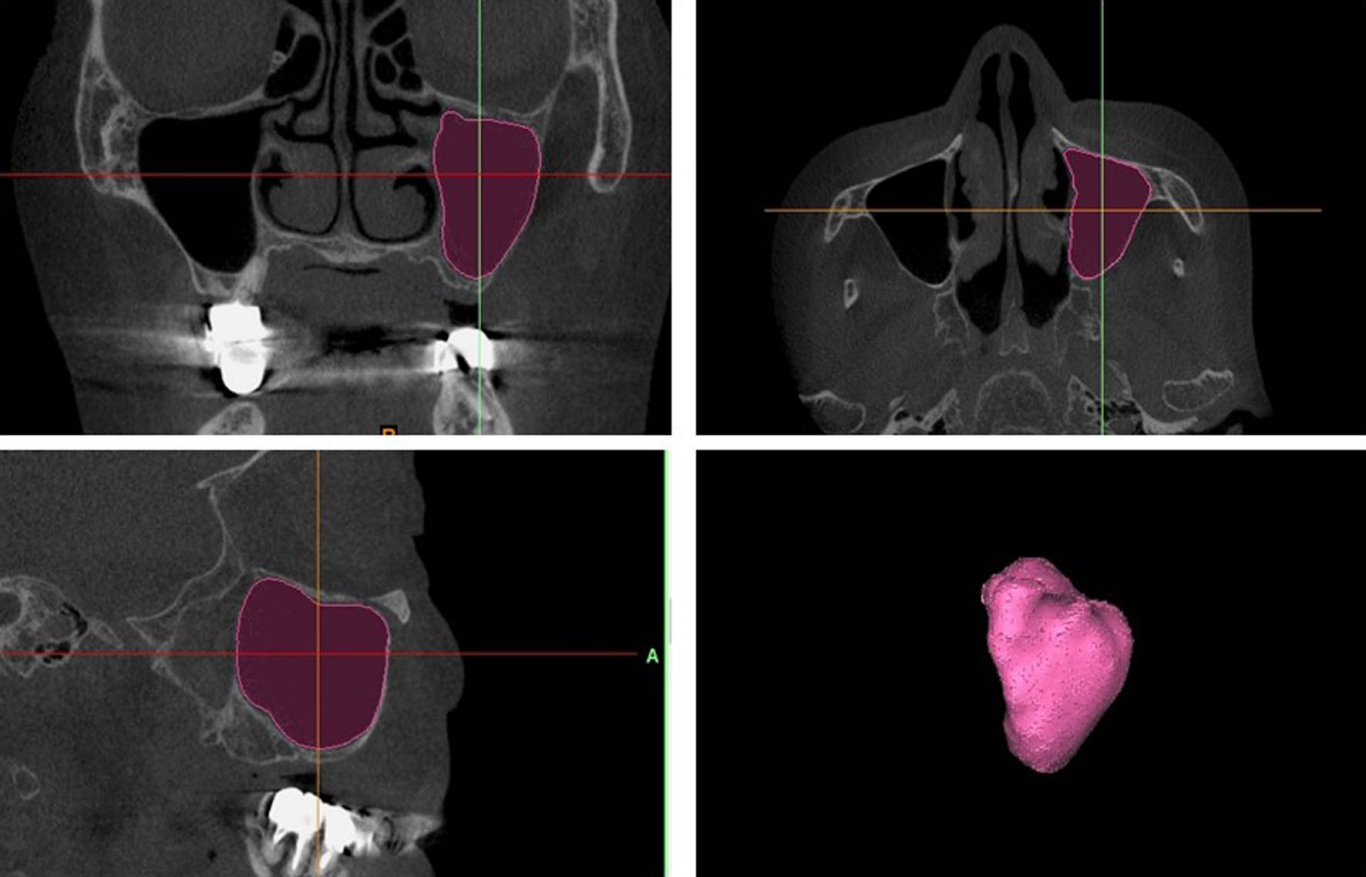
Method for measuring MSV. Upper-left: Visualization of the maxillary sinus in the coronal plane. Lower-left: Visualization of the maxillary sinus in the axial plane. Upper-right: Visualization of the maxillary sinus in the sagittal plane. Lower-right: Visualization of the maxillary sinus in three dimensions. *MSV* maxillary sinus volume

All images were obtained using the KaVo 3D eXam (KaVo Dental Systems, Biberach, Germany). The scanning parameters were 120 kVp, 5 mA, 8.9-s acquisition time, 0.25-mm-thick axial slice and isotropic voxel size, and imaging area of 16 × 16 cm. All images were recorded in the Digital Imaging and Communications in Medicine (DICOM) format. All data were composed of 0.25-mm axial slices as single DICOM files. Axial images were exported with a 512 × 512 matrix as a single frame for each DICOM file (Fig. 1).

The CBCT images were transferred to a computer, where the MSVs were measured using the MIMICS 23.0 software. Thresholding was applied with minimum and maximum limits of − 1024 HU and − 526 HU, respectively. The maxillary sinus was cropped using the software’s “edit masks” tool along the following borders: around the bone structure and narrowest space of the ostium between the infundibulum and the uncinate process. Subsequently, the connection with outer air was cropped slice by slice using the segmentation tools. Finally, the “region growing” tool was applied, enabling segmentation splitting created by thresholding into several objects. The MSVs were calculated using the software’s “calculate 3D” tool [12].

#### NSD Measurements

**Fig. 2:**
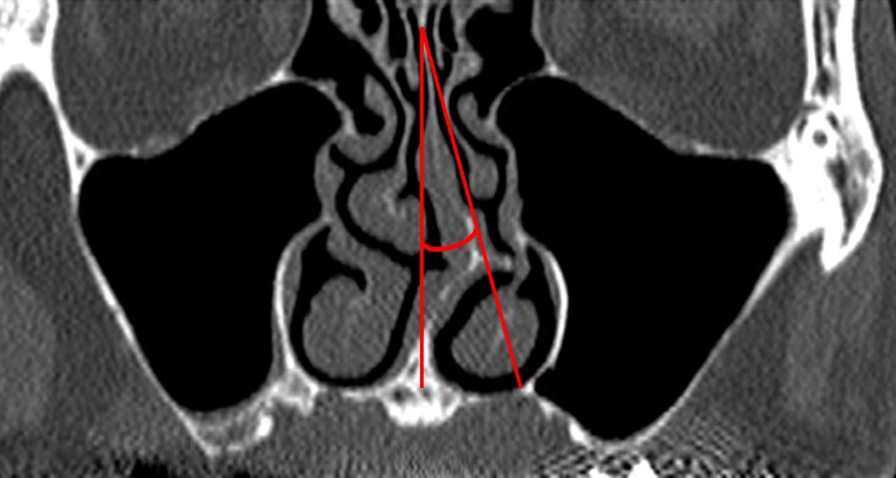
Method for measuring NSD. *NSD* nasal septal deviation

In accordance with our previous study [13], the presence of NSD was determined on coronal CBCT by the method described by Bhandary and Kamath [14]. A straight line was drawn from the maxillary anterior spine to the crista galli. The angle between the most defective part of the nasal septum and this straight line was measured and termed as the defection angle. A deviation greater than 9° denoted the presence of NSD and angles less than 9° denoted the absence of NSD (Fig. 2).

All imaging measurements based on CBCT were conducted by experienced dental radiology specialists. NSD was considered to be present if this deviation exceeded 9°.

### Statistical analysis

Due to large differences among patients, we compared the relationships of the presence/absence of teeth and NSD with MSV on the left and right sides within individual patients using the Wilcoxon *t*-test. *p*-values < 0.05 were considered statistically significant (PASW Statistics 18.0; SPSS Inc. SPSS, Japan).

## Results

The patients’ clinical characteristics are depicted in Table 1. The study population included 30 patients (60 sinuses; 12 men, 18 women). The average patient age was 58.2 ± 10.2 years (men, 60.4 ± 3.7 years; women, 59.2 ± 4.5 years; range, 40–77 years). The mean number of missing teeth was 2.98 ± 1.01: 13 patients had two missing teeth and 17 patients had three or more missing teeth. NSD was observed in 9 patients (30%).

**Table 1:** Patient data

		Men 12 women 18 (30 maxillary sinus)
Age		58.2 ± 10.2 years old
Number of missing teeth	Ave	2.98 ± 1.01 tooth
	Two tooth	13 persons
	More than three	17 persons
NSD	Present	9 persons
	Absent	2 l persons

The mean MSV on the non-edentulous side was 21.769 ± 4.30 cm^3^ (men: 23.01 ± 3.68 cm^3^, women: 21.050 ± 4.467 cm^3^), with a maximum value of 29.26 cm^3^ and a minimum value of 13.91 cm^3^. On the edentulous side, the mean MSV was 21.58 ± 3.890 cm^3^ (men: 23.246 ± 3.75 cm^3^, women: 20.62 ± 3.634 cm^3^), with maximum and minimum values of 28.37 cm^3^ and 11.74 cm^3^, respectively. In patients with NSD, the mean MSV on the ipsilateral and contralateral sides of the NSD was 22.098 ± 3.36 cm^3^ and 21.50 ± 3.84 cm^3^, respectively (Table 2). Thus, the MSV varied greatly among individual participants.

**Table 2:** MSV overall and by missing teeth, NSD, and sex

	Total	Missing tooth		NSD		Men		Women	
		Non-edentulous sides	Edentulous sides	Contralateral sides of the NSD	Ipsilateral sides of the NSD	Non-edentulous sides	Edentulous sides	Non-edentulous sides	Edentulous sides
Ave.	21.675	21.769	21.582	22.098	21.496	23.010	23.246	21.050	20.619
Median	21.796	22.122	21.676	21.999	21.087	23.702	24.140	21.529	21.061
Min	11.739	13.906	11.739	16.003	14.040	16.003	15.863	13.906	11.739
Max	29. 263	29.263	28.371	27.602	19.382	27.944	28.371	29.263	26.430
SD	4.10	4.300	3.890	3.559	3.840	3.677	3.753	4.467	3.634

*NSD* nasal septal deviation, *MSV* maxillary sinus volume

**Fig. 3:**
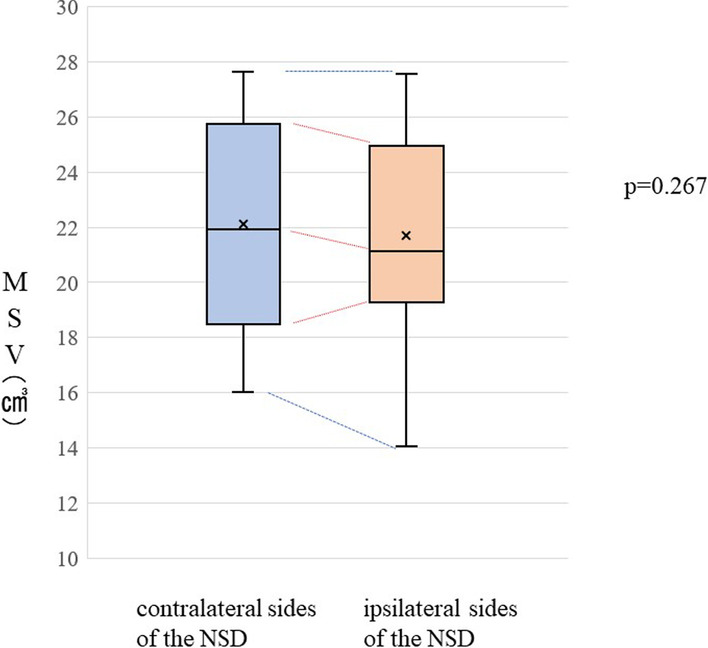
Comparisons of MSV based on the presence/absence of NSD. *NSD* nasal septal deviation, *MSV* maxillary sinus volume

Also, no implant loss was observed before the superstructure was placed. Intraoperative maxillary sinus mucosal perforation was observed in 2 patients (both had nasal septal deviation), and postoperative maxillary sinus inflammation was observed in 1 patient (this patient had nasal septal deviation, sinus volume: 24.146 cm^3^) (Fig. 3).

### Effect of NSD on MSV

The mean MSV on the ipsilateral and contralateral sides of the NSD was 21.50 ± 3.84 cm^3^ and 22.10 ± 3.56 cm^3^, respectively; thus, MSV did not differ significantly between the ipsilateral and contralateral sides (*p* = 0.150).

### Effect of missing teeth on MSV

**Fig. 4:**
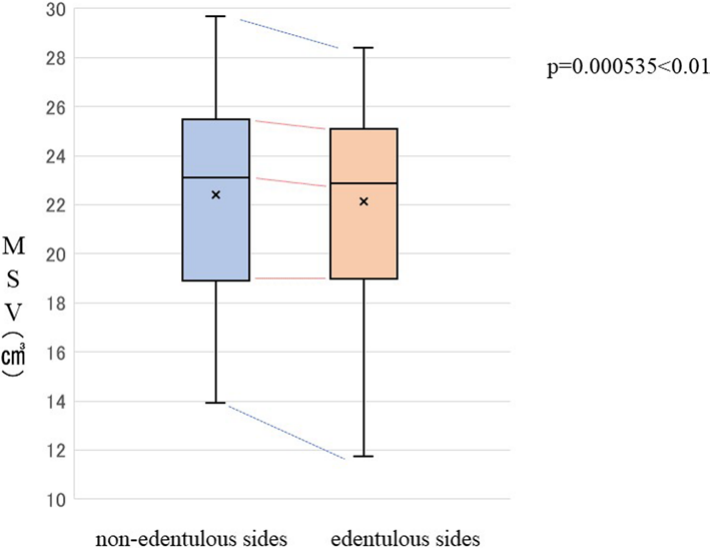
Comparisons of MSV based on missing teeth. *MSV* maxillary sinus volume

The mean MSV on the edentulous and non-edentulous sides was 21.58 ± 3.89 cm^3^ and 21.77 ± 4.30 cm^3^, respectively. Thus, MSV was significantly lower on the edentulous side than on the non-edentulous side (*p* = 0.00036) (Fig. 4).

## Discussion

The maxillary sinus is an important anatomical structure in maxillofacial surgery. Since previous studies have mainly focused on the condition of the maxillary sinus floor with respect to maxillary implants, there have been few reports on the effect of the presence or absence of a deviated nasal septum, polyp formation in the maxillary sinus, the presence or absence of a septum, the influence of bone head height on maxillary sinus volume [11, 15, 16], or the susceptibility to maxillary sinusitis due to a deviated nasal septum [7]. However, the maxillary sinus’ pathological state, shape, and size significantly affect the indications, difficulty, and rate of complications of maxillary sinus augmentation. Hence, assessing the maxillary sinus before dental implant treatment in the maxillary molar region is highly valuable for the operator.

The shape and size of the maxillary sinus are known to be affected by genetics, the environment, and other factors [17]. According to Kawarai et al. [18], paranasal sinus volume differs according to ethnicity and geography; Japanese people, in particular, have a larger MSV compared to other ethnicities. Additionally, a European study, conducted by Fernandes [19] who investigated European and Zulu populations, stated that the MSV was larger in the European population (mean: right side, 16.39 cm^3^; left side, 16.42 cm^3^) than in the Zulu population (mean: right side, 11.13 cm^3^; left side, 10.99 cm^3^). This study examined the MSV within individual patients due to the large ethnic and inter-individual variations described above.

The nasal septum comprises bone and cartilage that divide the nasal cavity bilaterally. A perfectly straight nasal septum is extremely rare, while the prevalence of NSD is reported to be 20–79% [14, 20]. Tao et al. stated that patients who develop sinusitis have highly deviated nasal septa [21]. Moreover, one study found that a high grade of deviation leads to obstruction of the OMC in the direction of deviation as well as frequent development and high severity of sinusitis [22]. As demonstrated by this study, the relationship between NSD and the maxillary sinus is absolutely crucial.

The current study found that the presence of NSD did not affect MSV. Orhan et al. [23] conducted a three-dimensional CT-based retrospective study on the relationship between NSD and MSV in 93 patients aged 16–79 years (mean age: 37.6 ± 14.4 years). They reported that the MSV was smaller on the ipsilateral side of the NSD than on the contralateral side. Similarly, Sapmaz et al. [24] and Kapusuz et al. [25] reported that NSD reduces the sinus volume on the ipsilateral side of the deviation. In contrast, Al-Rawi et al. [26] conducted a study on NSD and MSV in Arabic individuals (age range: 18 − 71 years; mean age: 39.25 ± 15.61 years) and did not observe a correlation between NSD and MSV. In a separate study of 209 patients with symptoms of sinusitis (mean age: 46.39 ± 17.17 years), Lee et al. [27] observed NSD in 50.48% of patients but did not observe a correlation between NSD and MSV.

In this study, the prevalence of NSD was low, and MSV did not differ between the ipsilateral and contralateral sides of the deviation, conceivably because we examined MSV in patients aged ≥ 40 years and excluded patients with a history of paranasal sinus disease.

Maxillary sinus floor pneumatization is generally said to occur following maxillary molar extraction [28]. It is commonly considered that as teeth are lost, both alveolar resorption and the maxillary sinus pneumatization lead to its expansion [28–30].

In previous studies using CBCT and CT, two-dimensional anteroposterior and mediolateral measurements were taken as the sinus dimension, and it has been suggested that the influence of maxillary sinus pneumatization is greater than that of alveolar bone resorption with respect to the influence of tooth loss and age. However, in the current study, three-dimensional evaluation (using software) of SMV was investigated.

Regarding the effect of age, in a 3D evaluation study, Aktuna et al. [12] examined the effect of age on patients with all permanent teeth and reported that maxillary sinus volume decreases with age increase. In addition, Velasco-Torres et al. [31] reported that volume decreases with age increase. Regarding the effect of tooth loss, also in a 3D study, Luz et al. [32] reported that there is no relationship between tooth loss and MSV using comparisons by dentition state (edentulous, partly edentulous, dentate). Moreover, Scriber et al. [33] conducted a similar study and reported no effect of tooth loss on sinus pneumatization.

Conversely, Velasco-Torres et al. found that maxillary sinus volume was smaller in completely and partially edentulous patients than in dentate patients which they cited as being due to the lack of stimulation of the maxillary bone [31]. Similarly, Möhlhenrich et al. [34] reported that MSV decreases with increase in tooth loss due to the decrease in bone stress. In this study, a comparison of the MSV on the edentulous and non-edentulous sides within individual patients revealed that MSV was significantly lower on the edentulous side (*p* < 0.005). Although we examined unilateral partially edentulous patients to compare MSV within each individual, our results were similar to those of Velasco-Torres et al. [31] and Möhlhenrich et al. [34].

This study differs from other studies in that we compared differences in sinus volume due to tooth loss between the same patients. Therefore, we postulated that the sinus volume decrease was not due to the influence of age. Rather, this decrease was due to the influence of lack of stimulation of the maxillary bone. Furthermore, although it was not part of the inclusion criteria, the fact that the tooth extraction timing of all the target patients was within one year after tooth extraction may have had some influence on the results of this study.

This study was a pilot investigation that only examined maxillary sinus augmentation using preoperative CT. The study limitations were as follows. First, we did not examine the association between MSV and postoperative complications. Second, we could not assess the effect of the number of missing teeth due to the small sample size. Third, we did not examine the relationships between MSV and types of ostial obstruction other than NSD. In the future, we endeavor to use larger sample sizes and examine the associations of MSV with other forms of ostial obstruction, membrane thickness, anatomical structures in the maxillary sinus (including sinus septa), and postoperative complications, such as sinusitis.

## Conclusion

MSV was not associated with NSD but was greatly reduced due to missing teeth using three-dimensional CBCT measurements of the maxillary sinuses in partially edentulous patients.

## Data Availability

The datasets of the current study are available from the corresponding author on reasonable request.

## Abbreviations

CBCT: Cone-beam computed tomography
CT: Computed tomography
MDCT: Multidetector computed tomography
NSD: Nasal septal deviation
OMC: Ostiomeatal complex
PA: Periapical lesion
MSV: Maxillary sinus volume
